# Assessment of Vehicle Volatility and Deposition Layer Thickness in Skin Penetration Models

**DOI:** 10.3390/pharmaceutics13060807

**Published:** 2021-05-28

**Authors:** Abdullah Hamadeh, John Troutman, Andrea N. Edginton

**Affiliations:** 1 School of Pharmacy, University of Waterloo, Kitchener, ON N2G 1C5, Canada; ahamadeh@uwaterloo.ca; 2 The Procter & Gamble Company, Mason, OH 45040, USA; troutman.ja@pg.com

**Keywords:** dermal, skin, permeation, in silico, models, vehicle, volatility

## Abstract

Systemic disposition of dermally applied chemicals is often formulation-dependent. Rapid evaporation of the vehicle can result in crystallization of active compounds, limiting their degree of skin penetration. In addition, the choice of vehicle can affect the permeant’s degree of penetration into the stratum corneum. The aim of this study is to build a predictive, mechanistic, dermal absorption model that accounts for vehicle-specific effects on the kinetics of permeant transport into skin. An existing skin penetration model is extended to explicitly include the effect of vehicle volatility over time. Using in vitro measurements of skin penetration by chemicals applied in both a saline and an ethanol solvent, the model is optimized to learn two vehicle-specific quantities: the solvent evaporation rate and the extent of permeant deposition into the upper stratum corneum immediately following application. The dermal disposition estimates of the trained model are subsequently compared against those of the original model using further in vitro measurements. The trained model showed a 1.5-fold improvement and a 19-fold improvement in overall goodness of fit among compounds tested in saline and ethanol solvents, respectively. The proposed model structure can thus form a basis for in vitro to in vivo extrapolations of dermal disposition for skin formulations containing volatile components.

## 1. Introduction

Establishing reliable estimates of the bioavailability of dermally applied chemicals is a requirement for efficacy and risk assessment studies and for subsequent regulatory approval. Bioavailability may be inferred by training an in silico model of dermal absorption using in vitro skin permeation test data and then extrapolating the trained model to predict the disposition of actives in the in vivo setting [1]. The reliability of such model-based approaches, however, depends on a quantitative understanding of the processes that ultimately determine dermal absorption.

Following application of a dose preparation to the skin surface, the formulation components begin to undergo a series of transport processes: (1) a fraction of the applied dose on the skin surface permeates into a ‘deposition layer’ occupying the upper stratum corneum (SC) through a process of convection [2], (2) the concentration difference between the vehicle and the top layers of the SC drives diffusion of the permeant [3], and, (3) depending on exposure and ambient conditions, volatile components of the formulation may evaporate. While evaporation of the solvent can concentrate active ingredients near the skin surface, accelerating absorption, the eventual precipitation of active ingredients on the skin surface can inhibit their diffusive flux into the SC [4,5,6,7]. The time scales over which these processes occur have significant bearing on the degree of cumulative skin penetration and, importantly, are often formulation-dependent [8].

In the earlier modeling work reported in Dancik et al. [9], the vehicle/stratum corneum boundary conditions of Kasting and Miller [2,10] were integrated with the skin layer partitioning, diffusion and clearance models reported in [11,12,13,14]. Among the assumptions of the Dancik et al. [9] model is that, in the presence of a solvent, the deposition layer extends to the upper tenth of the stratum corneum (SC), irrespective of the solvent used. If the solvent is volatile, any portion of an applied dose in excess of the deposition layer capacity is assumed to settle on the skin surface and, from there, be gradually absorbed.

The aim of this study is to develop, with the aid of experimental data, a mechanistic model of skin penetration that can form the basis of in vitro to in vivo extrapolations of chemicals that are dermally applied using volatile vehicles. We demonstrate the need for the inclusion of vehicle-specific evaporation rates and deposition layer depths in such models by comparing the dermal disposition estimates generated by the Dancik et al. [9] model against measurements from sixty-one in vitro permeation tests (IVPTs) conducted in Hewitt et al. [5] using multiple solvents. The main objectives are: (1) to develop a mechanistic model of dermal absorption that accounts for vehicle-specific effects in the kinetics of the transport processes that deliver the permeant into the stratum corneum, (2) to train the model using experimental data from Hewitt et al. [5] that report the dermal disposition of chemicals when applied to skin, un-occluded, in saline and in ethanol solvents, (3) to assess the trained model by comparing its dermal disposition with further measurements of in vitro skin permeation reported in Hewitt et al. [5] for thirty-one compounds. 

As an outcome of this work, we propose a framework for future inference of in vivo dermal absorption given IVPT data. In addition, we outline future research directions in which the approach adopted in this study may be extended to enable the model-based selection of formulation candidates that are likely to yield target levels of dermal delivery of novel active ingredients for efficacy and safety assessments. 

## 2. Materials and Methods

### 2.1. Two Models of Penetration into the Stratum Corneum

We summarize two models of the transport of permeants from simple volatile vehicles into the SC based on Dancik et al. [9]. Model nomenclature is presented in Appendix A and mathematical details of the two models are presented in Appendix B.

When a permeant compound is applied to the skin surface, both models hypothesize that a portion of the dose is convected into a deposition layer at the top of the SC that occupies a proportion $\eta_{dep}$ of the total SC thickness $h_{sc}$ [2]. If the applied mass per area $M_{0}$ is below the deposition layer’s saturation capacity ($M_{sat}$), the dose is assumed to be entirely absorbed into this sub-compartment (and therefore, into the SC). In cases where the dose exceeds the capacity of the deposition layer, the excess permeant (of mass per area $M_{surf}$) is deposited on the skin surface. The capacity of the deposition layer is given by the expression (Equation (1)):

(1) $$M_{sat}=\eta_{dep}h_{sc}C_{sat}$$

where $C_{sat}$ is the saturating concentration of the stratum corneum given by $C_{sat}=K_{sc/w}S_{w}$, $K_{sc/w}$ is the SC/water partition coefficient and $S_{w}$ is the solubility of the permeant in water. The partition coefficient $K_{sc/w}$ is given in Wang et al. [12] and is a function of the lipophilicity of the permeant and the lipid volume fraction of the SC. Under these assumptions, following an initial permeant application, the initial mass per area on the skin surface is given by (Equation (2)):

(2) $$M_{surf}\left(0\right)=\max\left(0,M_{0}-\eta_{dep}h_{sc}C_{sat}\right)$$

and the permeant concentration in the stratum corneum $c_{sc}\left(z,t\right)$ within the deposition layer is, at time t=0 (Equation (3)):

(3) $$c_{sc}\left(z,0\right)=\min\left(\frac{M_{0}}{\eta_{dep}h_{sc}},C_{sat}\right),\;0\leq z<\eta_{dep}h_{sc}$$

whereas the initial SC permeant concentration is assumed to be zero beneath the deposition layer (Equation (4)):

(4) $$c_{sc}\left(z,0\right)=0,\;\eta_{dep}h_{sc}\leq z<h_{sc}$$

#### 2.1.1. Model A

This model is the ‘volatile vehicle’ (Cases 1 and 2) model of Dancik et al. [9] and assumes that $\eta_{dep}=0.1$ when the permeant is applied in a solvent. This model also assumes the boundary condition in Kasting and Miller [2] under which, if a dose large enough to saturate the deposition layer is applied, the saturated state is maintained by balancing the permeant flow from the skin surface into the deposition layer with any flow out of the deposition layer. Formally, this boundary condition is given by Equation (5)):

(5) $${\dot{M}}_{surf}=\{\begin{array}{rr} -k_{evap-per}-D_{sc}{\frac{\partial c_{sc}\left(z,t\right)}{\partial z}}_{|z=\eta_{dep}h_{sc}}, & M_{surf}>0\\ 0, & \mathrm{otherwise} \end{array}\;$$

where $D_{sc}$ is the SC diffusion coefficient, as modeled in [9], and where $k_{evap-per}$ is the evaporation rate of the permeant. This rate is given by equations 36 and 37 in [2] and is a function of wind velocity (denoted u).

#### 2.1.2. Model B

This model makes no prior assumption on the deposition layer proportion of the SC, $\eta_{dep}$. We denote the volatile vehicle’s thickness at time t by $h_{v}\left(t\right)$, which has an initial value $h_{v}\left(0\right)$ and which varies over time, due to evaporation, according to the zeroth-order process (Equation (6)):

(6) $${\dot{h}}_{v}=\{\begin{array}{c} -k_{evap-veh},\\ 0, \end{array}\begin{array}{r} h_{v}>0\\ \mathrm{otherwise} \end{array}$$

where $k_{evap-veh}$, the evaporation rate of the vehicle, is assumed constant.

The mass per area of the permeant on the skin surface $M_{surf}$ follows the model (Equation (7)):

(7) $${\dot{M}}_{surf}=\{\begin{array}{rr} -k_{evap-per}-D_{sc}{\frac{\partial c_{sc}\left(z,t\right)}{\partial z}}_{|z=0}, & h_{v}>0\;\mathrm{and}\;M_{surf}>0\\ -k_{evap-per}, & h_{v}=0\;\mathrm{and}\;M_{surf}>0\\ 0, & \mathrm{otherwise} \end{array}$$

Under this model, the skin surface permeant is gradually depleted either by evaporation or by diffusion into the stratum corneum, and diffusion only occurs in the presence of the vehicle ($h_{v}>0$). We assume that only the portion of the permeant dissolved in the vehicle is capable of diffusing into the SC. The concentration of the permeant in the vehicle is given by $c_{v}=\min\left(S_{v},\;M_{surf}/h_{v}\right)$, where $S_{v}$ is the maximum solubility of the permeant in the vehicle. As in Cases 3 and 4 in Dancik et al. [9], the vehicle/stratum corneum partition coefficient is given by $K_{v/sc}=K_{v/w}/K_{sc/w}$, where $K_{v/w}=S_{v}/S_{w}$ is the vehicle/water partition coefficient. 

In summary, three features differentiate Model B from Cases 3 and 4 in Dancik et al. [9]: (i) the vehicle evaporates over time, (ii) as it does so, the permeant precipitates out of solution once its concentration exceeds $S_{v}$, and (iii) once the vehicle has evaporated completely, diffusion of the permeant into the SC ceases as observed in Chiang et al. [4], Oliviera et al. [6] and Akhter and Barry [7].

### 2.2. IVPT Data

Hewitt et al. [5] report the results of 61 IVPTs conducted on 56 cosmetically relevant small molecule compounds (MW < 500 Daltons). The selected compounds include ionizable and non-ionizable permeants with a wide range of molecular weights (92 to 371 g/mol), melting points (−90 to 300 degrees C), and log P values (−1.42 to 4.76). 

Each IVPT was conducted on three replicate skin sections from each of four skin donors, under non-occluded conditions, over a 24 h period. A phosphate buffered saline (PBS) solvent was used in all but eleven IVPTs, ten of which were applied using an ethanol solvent and one using acetone. 

Four compounds (geraniol, benzophenone, propylparaben and hydrocortisone) were tested in both a PBS and an ethanol solvent. Of the 61 IVPTs, 20 were conducted in a fume hood. 

The reported quantities were the remnant permeant on the skin surface after 24 h (the ‘skin wash’), the accumulated permeant mass in the receptor fluid at multiple timepoints over the 24 h period, and the accumulated permeant mass at 24 h in each of the SC, epidermis, dermis, and receptor fluid. 

### 2.3. Assessment of Model A Deposition Layer Capacity

As a preliminary assessment of Model A, the theoretical capacity of the deposition layer proposed in Dancik et al. [9] was calculated for each of the sixty-one IVPTs in Hewitt et al. [5] using Equation (1) and then compared to the applied dose and remaining skin wash at 24 h. In all IVPTs, the permeant was applied in either a PBS, ethanol, or acetone solvent, and therefore the depth of the deposition layer was taken to be a tenth of the stratum corneum thickness ($\eta_{dep}=0.1$), as per Dancik et al. [9]. The SC thickness was assumed to be 13 µm, corresponding to a partially hydrated SC [9]. This assumption is made because all skin samples [5] had been dried ahead of permeant application and because the applied volume, 10 µL, was unlikely to fully hydrate the SC in the case of aqueous solvents. The saturating concentration of the SC, $C_{sat}$, was evaluated for each compound separately via equation C.11 in Dancik et al. [9] using the lipophilicities and water solubilities provided in Hewitt et al. [5].

### 2.4. Optimization and Assessment of Predictive Performance of Models A and B

Prior to assessing the predictive performance of Models A and B, their uncertain parameters were calibrated using observations from six IVPTs from [5] that were conducted on three compounds in both PBS and ethanol solvents: benzophenone, propylparaben (experiment 1 in [5]) and hydrocortisone. A fourth compound that was tested in [5] in both PBS and ethanol, geraniol, was not used in the optimization due to its low mass balance at 24 h. 

Following optimization, the predictive performance of the two models was assessed against observed data from additional IVPTs reported in Hewitt et al. [5]. Since the present aim is to determine the impact of the deposition layer and vehicle volatility using Models A and B, we control for the effect of permeant volatility by limiting the comparative assessment of the two models to thirty-one IVPTs from Hewitt et al. [5] that were not conducted in a fume hood and in which the mass balance exceeded 80% at the end of the 24-h experiment. Of these 31 IVPTs, 26 were conducted using a PBS solvent (listed in Table 1) and five using a pure ethanol solvent (listed in Table 2).

#### 2.4.1. Optimization and Assessment of Model A

All IVPTs used for model optimization and assessment were not conducted in a fume hood. We therefore assume that all experiments shared a common, unknown, ambient wind velocity u. According to the permeant evaporation model in [2], this parameter directly impacts the permeant evaporation rate $k_{evap-per}$. As such, it is a parameter to which the permeant mass balance at the conclusion of the IVPT (as defined in Table A3) is sensitive. Thus, a log-likelihood function for u in Model A ($u_{A}$) was taken to be the negated sum of the square of mass balance errors $E_{MB}$ (defined in Table A4) at t=24 h, summed over the IVPTs conducted in PBS and ethanol solvents for benzophenone, propylparaben (experiment 1) and hydrocortisone in [5]. The maximum likelihood estimate of $u_{A}$ was obtained by maximizing this function. 

Following calibration of $u_{A}$, Model A was assessed via the error metrics defined in Table A4, evaluated at t=24 h, for each IVPT in Table 1 and Table 2. For a given IVPT, these metrics quantify the overall performance of the model via the total weighted sum of squares error, $E_{Total}^{2}$, and they assess the model’s ability to predict permeant accumulation in the different skin model compartments via the skin wash error $e_{SW}^{2}$, the stratum corneum accumulation error $\left(e_{SC}^{2}\right)$ and the cumulative dermal delivery error $\left(e_{DD}^{2}\right)$.

#### 2.4.2. Optimization and Assessment of Model B

As with Model A, the wind velocity u impacts the Model B estimate of the mass balance at the conclusion of the IVPT, which equates to the total permeant that remains on the skin surface, accumulates in the SC, or is dermally delivered at t=24 h. In addition, with respect to Model A, Model B introduces a vehicle evaporation rate $k_{evap-veh}$ and assumes that the deposition layer size $\eta_{dep}$ can differ from the value of 0.1 proposed in [9]. Since $k_{evap-veh}$ impacts the time at which dermal absorption ceases due to vehicle evaporation, it also impacts the model’s estimate of the skin wash, stratum corneum accumulation and dermal delivery at the conclusion of the IVPT. These outputs are additionally sensitive to $\eta_{dep}$, which determines the degree of SC penetration at the start of the experiment. Due to the different volatilities of the PBS and the ethanol solvents [5] and the reported role of ethanol as a penetration enhancer [15], it was assumed that $k_{evap-veh}$ and $\eta_{dep}$ are vehicle-specific parameters. 

Model B was optimized by maximizing the likelihood of $\eta_{dep}$ for PBS and ethanol solvents (respectively denoted $\eta_{dep_{B}}^{pbs}$ and $\eta_{dep_{B}}^{ethanol}$), the vehicle evaporation rates $k_{evap-veh}$ for PBS and ethanol solvents (respectively denoted $k_{evap-veh}^{pbs}$ and $k_{evap-veh}^{ethanol}$) and the wind velocity u (denoted $u_{B}$). The log-likelihood function was taken to be the negated total weighted sum of squares error, $E_{Total}^{2}$, between Model B outputs and their respective observations from the six IVPTs conducted on benzophenone, propylparaben (experiment 1) and hydrocortisone in [5]. The model outputs were the skin wash, stratum corneum accumulation and dermal delivery at 24 h. Following optimization, the predictive performance of Model B was assessed as with Model A, through evaluation of $E_{Total}^{2}$, $e_{SW}^{2}$, $e_{SC}^{2}$ and $e_{DD}^{2}$ for all thirty-one IVPTs in Table 1 and Table 2.

### 2.5. AIC Analysis

One parameter, the wind velocity $u_{A}$, was calibrated for Model A, whereas five parameters ($\eta_{dep}^{pbs}$, $\eta_{dep}^{ethanol}$, $k_{evap-veh}^{pbs}$, $k_{evap-veh}^{ethanol}$, *μ_B_*) were calibrated for Model B. Model B therefore benefits from four additional degrees of freedom with respect to Model A. The Akaike information criterion [16] was used to assess the relative goodness of fit of Model B compared to Model A given these additional calibration parameters.

### 2.6. Software

Prior to this study, the skin permeation model reported in Dancik et al. [9] had been programmed into MoBi, part of the Open Systems Pharmacology Suite v9 (www.open-systems-pharmacology.org, accessed on 31 March 2021). This open-source implementation of the model is available on GitHub [17] and was used for all simulations of Model A, while a modified version of this model was used to execute simulations of Model B. Optimization of the models was performed in R by applying the Nelder-Meade algorithm [18] of the R package nloptr to the MoBi models via the OSPSuite-R package, which is also part of the Open Systems Pharmacology Suite.

## 3. Results

### 3.1. Assessment of Model A Deposition Layer Capacity

For each of the IVPTs tested in Hewitt et al. [5], Table A5 in Appendix C lists the applied permeant dose and measured skin wash at 24 h along with the Model A deposition layer capacity $M_{sat}$. There was a detectable amount of permeant in the skin wash of all IVPTs. In 37 of those 61 experiments, the deposition layer capacity exceeded the dose, indicating that the deposition layer absorbed less than is predicted by Model A.

### 3.2. Optimization of Models A and B

**Figure 1:**
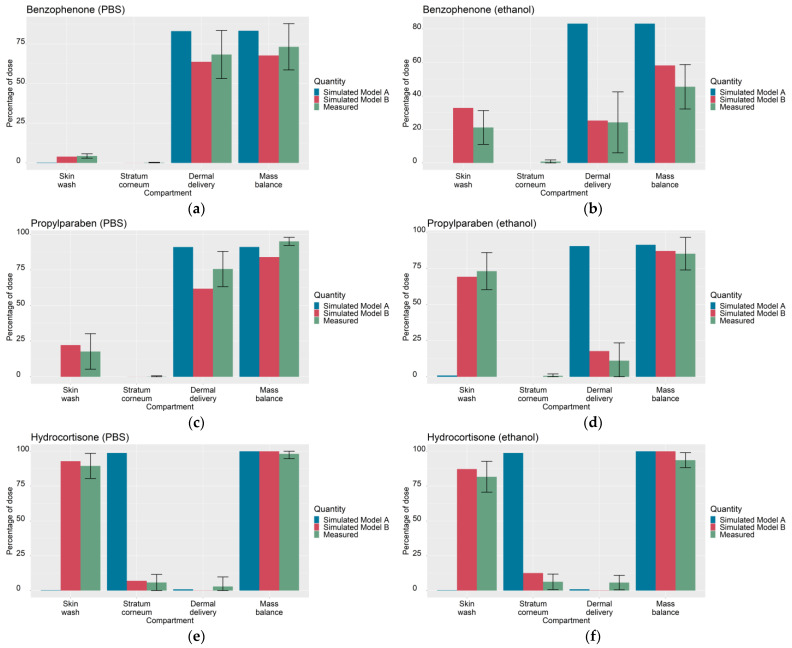
Simulated outputs of optimized Models A and Model B (skin wash, stratum corneum accumulation, cumulative dermal delivery and mass balance at 24 h) and corresponding experimental measurements (from [5]) used to calibrate (for Model A) the wind velocity $u_{A}$ and (for Model B), the wind velocity $u_{B}$, the deposition layer proportions of the SC ($\eta_{dep}^{pbs}$, $\eta_{dep}^{ethanol}$) and the vehicle evaporation rate ($k_{evap-veh}^{pbs}$, $k_{evap-veh}^{ethanol})$ under PBS and ethanol solvents; (**a**) benzophenone in PBS, (**b**) benzophenone in ethanol, (**c**) propylparaben in PBS, (**d**) propylparaben in ethanol, (**e**) hydrocortisone in PBS, (**f**) hydrocortisone in ethanol. The total root sum of squares error, defined in Table A4, is 252.5 for Model A and 190.4 for Model B.

The maximum likelihood estimates of the optimized parameters for Models A and B are summarized in Table 3. As a visual predictive check, Figure 1 shows the optimized outputs of Models A and B and the corresponding experimental measurements for the six IVPTs to which the models were optimized. 

### 3.3. Assessment of Models A and B

**Figure 2:**
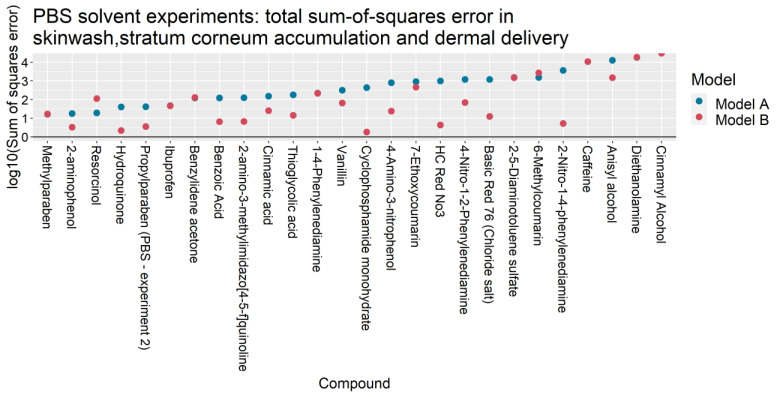
Total sum-of-squares errors $E_{Total}^{2}$ (as defined in Table A4) of Models A and B for IVPTs conducted using PBS solvents.

**Figure 3:**
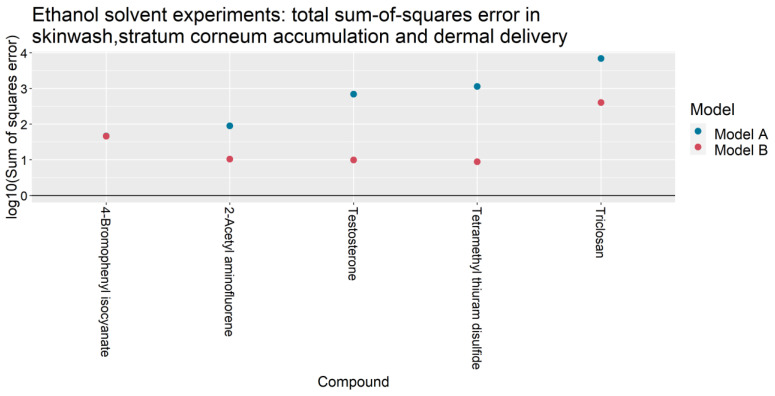
Total sum-of-squares errors $E_{Total}^{2}$ (as defined in Table A4) of Models A and B for IVPTs conducted using a pure ethanol solvent.

For the 31 IVPTs selected for model assessment, Table 4 compares the skin wash, SC accumulation and dermal delivery at 24 h, as predicted by Models A and B, against the experimental values from Hewitt et al. [5]. For each experiment, the predicted quantities were generated after updating Models A and B with their respective optimized parameter values in Table 3. For each IVPT, the weighted sum of squares of the model errors $E_{\mathrm{Total}}^{2}$ (defined in Table A4) are plotted in Figure 2 and Figure 3 for IVPTs that used PBS and ethanol solvents respectively. The error in the Model B estimate was found to be lower than that of Model A in 20 of the 26 IVPTs conducted in a PBS solvent, and in all five IVPTs conducted in the pure ethanol solvent. 

An AIC difference of −55,041 was found in favor of Model B, indicating a significant improvement in goodness of fit that overcomes the penalty for the loss of model parsimony due to four additional parameters with respect to Model A.

Table 5 presents the sum of squares errors for the different model outputs summed over the IVPTs used for model assessment in each solvent. For IVPTs conducted using PBS solvents, Model A yielded a smaller error than Model B in predicting the skin wash and dermal delivery at 24 h, whilst Model B better predicted the stratum corneum accumulation. Model B yielded a significantly lower error in all model outputs among IVPTs conducted in ethanol.

**Figure 4:**
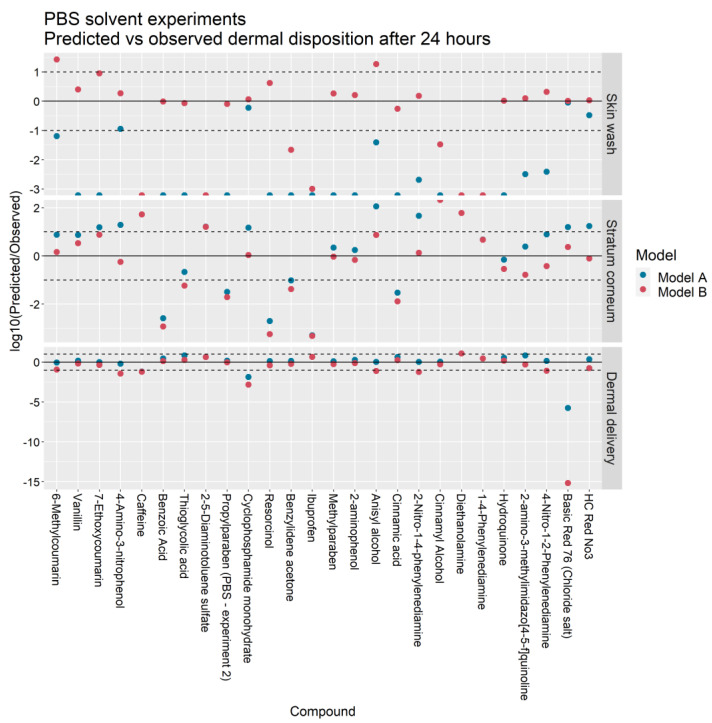
Predicted vs. observed skin wash, stratum corneum accumulation and dermal delivery in IVPTs conducted in a PBS solvent for Models A and B. Points between the dashed lines indicate predictions that are within one order of magnitude of observations.

**Figure 5:**
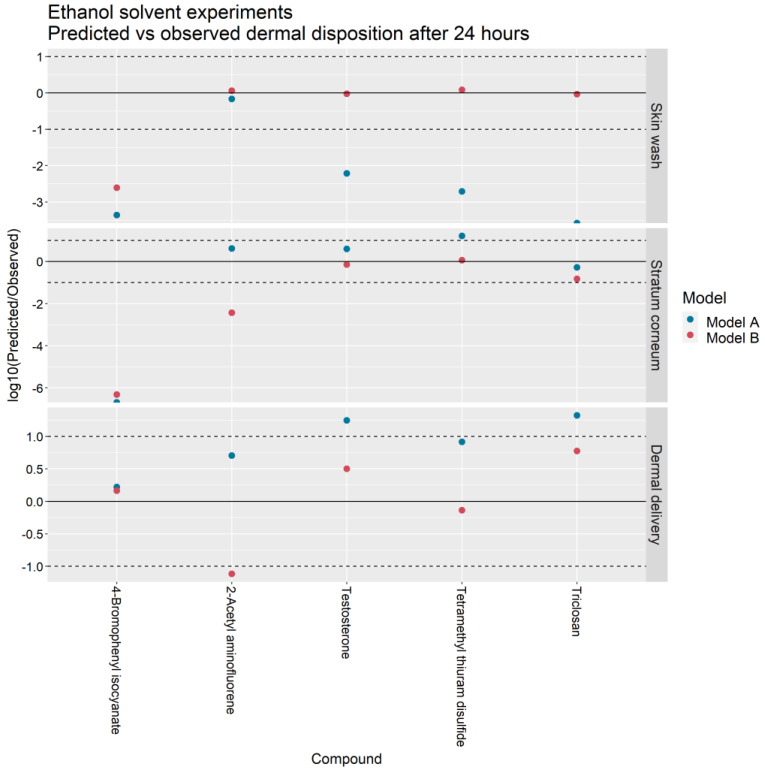
Predicted vs. observed skin wash, stratum corneum accumulation and dermal delivery in IVPTs conducted in an ethanol solvent for Models A and B. Points between the dashed lines indicate predictions that are within one order of magnitude of observations.

Figure 4 and Figure 5 illustrate the ratio of the predicted outputs of Models A and B to the corresponding mean observations from Hewitt et al. [5]. With respect to Model A, the skin wash at 24 h predicted by Model B more closely matched the IVPT measurements in 25 of the 26 PBS solvent experiments and 4 of the 5 ethanol solvent experiments. The stratum corneum accumulation at 24 h was better predicted by Model B in 15 of the 26 PBS experiments and 3 of the 5 ethanol experiments. The cumulative dermal delivery at 24 h was better predicted by Model B in 11 of the 26 PBS experiments and 4 of the 5 ethanol experiments.

For the skin wash, SC accumulation and dermal delivery, the Model A predictions were within ten-fold the experimental value in 4/26, 9/26 and 22/26 of the PBS solvent experiments and in 1/5, 3/5, and 3/5 of the ethanol solvent experiments. In contrast, the Model B predictions were within ten-fold the experimental value in 17/26, 15/26 and 18/26 of the PBS experiments and in 4/5, 3/5, and 4/5 of the ethanol experiments.

In terms of dermal delivery, Model A overpredicted the experimental value in 20/26 PBS solvent experiments and 5/5 ethanol experiments whereas Model B overpredicted the experimental value in 8/26 PBS experiments and 3/5 ethanol solvent experiments.

## 4. Discussion

The process of dermal absorption is, in general, highly sensitive to the permeant’s physical and chemical parameters, ambient conditions and the choice of excipient [6,19]. Samaras et al. [20] showed that the volatility of the vehicle was a key factor influencing permeant flux, while Davis et al. [21] reported the role of solvents in enhancing the permeation into the SC.

Earlier work by Krüse et al. [1] demonstrated the feasibility of training a mechanistic dermal absorption model with infinite dose IVPT data and subsequently extrapolating the trained model to predict skin penetration under finite dose scenarios. In contrast, the focus of the present study has been to learn vehicle-specific parameters of Model B from in vitro experiments that measure the dermal absorption of multiple compounds applied in common excipients: PBS and ethanol (Figure 1). These vehicle-specific parameters were the vehicle volatility $k_{evap-veh}$, which limits the degree of permeant diffusion into the SC, and the depth of the deposition layer in the SC, $\eta_{dep}$. Based on these learned quantities, the trained model was then extrapolated to predict the dermal absorption of thirty-one compounds applied in PBS and ethanol vehicles (Figure 4 and Figure 5).

We next discuss the main findings of this study and then compare the dermal disposition estimates generated by Model A and Model B.

### 4.1. Model A Overpredicts the Capacity of the Deposition Layer

In the preliminary assessment of Model A, the capacity of the deposition layer with respect to each compound was evaluated and compared to the applied dose and the observed skin wash at 24 h. In the foundational work by Kasting and Miller [2], the deposition layer is theorized to absorb at least a portion of the dose immediately upon application through convection, effectively providing a shortcut into the SC, especially for compounds that have a low SC/vehicle partition coefficient. In theory, this portion can extend to the entirety of the dose when the applied amount is exceeded by the deposition layer’s capacity.

The estimated capacity of the deposition layer is determined by its depth and the solubility of the applied permeant in the SC. Based on earlier studies on DEET by Kasting et al. [22], Model A assumes that the deposition layer depth equates to a tenth of the SC thickness $h_{sc}$, and is given by $\eta_{dep}h_{sc}$, where $\eta_{dep}=0.1$. As shown in Appendix C, there was a detectable skin wash among all thirty-seven (out of 61) experiments in which the dose was smaller than the hypothesized deposition layer capacity. This capacity is therefore likely to be an overestimate. Since this overestimate was observed in both hydrophilic (caffeine) and lipophilic (ibuprofen) compounds, the excess capacity of this layer is most likely due to an overestimate of the deposition layer depth, and not SC solubility.

The excess capacity of this layer, and the resulting overestimate of permeant absorption partly explains why Model A systematically underestimated the skin wash and over-estimated the dermal delivery. In Model B, the optimized value of $\eta_{dep}$ was found to be 0.03 in the case of PBS solvents and 0.05 in the case of pure ethanol solvents, thus demonstrating the role of the latter as a penetration enhancer [15].

### 4.2. Vehicle Volatility Limits Permeant Diffusion into the SC

**Figure 6:**
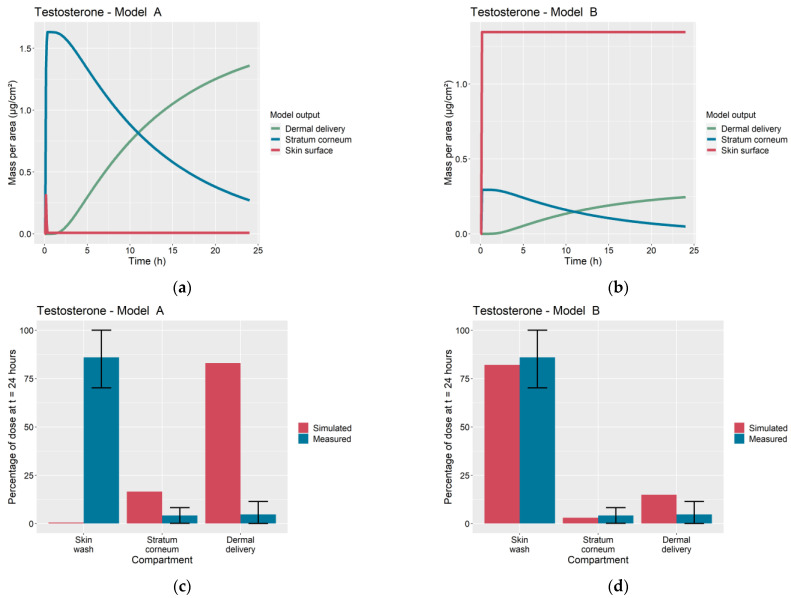
Comparison of Model A and Model B simulations of dermal absorption of testosterone. Time profiles of permeant mass per area on skin surface, accumulated in stratum corneum and dermally delivered for (**a**) Model A and (**b**) Model B. Simulated and measured percentage of dose remaining in skin wash, accumulated in stratum corneum and dermally delivered at 24 h for (**c**) Model A, root sum of squares error (Table A4) $E_{Total}^{2}=615.5$, and (**d**) Model B, $E_{Total}^{2}=7.77$.

The overprediction of dermal delivery by Model A is additionally attributed to continued diffusion of the permeant into the SC over the course of the 24 h of the IVPT. In contrast, Model B assumes that diffusion ceases following complete evaporation of the solvent, as has been observed experimentally in [4,5,6,7]. This qualitative difference in the kinetics of permeant absorption is illustrated in Figure 6a,b for the case of the testosterone IVPT from Hewitt et al. [5], which used an ethanol solvent. Shortly after application, Model A predicts a rapid diffusion of the entire permeant dose on the skin surface into the SC, followed by slow dermal delivery. In Model B, there is virtually no diffusion into the SC from the vehicle due to the fast evaporation of the ethanol solvent. Here, the portion of the dose that does enter the SC is essentially limited to that absorbed into the deposition layer upon application. A comparison of Figure 6c with Figure 6d shows that Model B is better able to explain the experimental data from [5]. For dermal applications involving volatile vehicles, this figure illustrates the importance of quantifying the degree of permeant absorption into the SC over short timescales at the beginning of the experiment.

### 4.3. Model B Predicts Greater Permeant Accumulation on the Skin Surface

Figure 4 and Figure 5 show that Model B predicts a greater proportion of the applied dose remaining on the skin surface at the conclusion of the IVPTs than Model A. This effect can be attributed to Model B’s smaller deposition layer and early cessation of permeant absorption due to vehicle volatility. In addition, this effect can explain the significant difference in the estimated wind velocities $u_{A}$ and $u_{B}$ for the two models (Table 3): to achieve the correct mass balance, Model A needs to reach a greater degree of permeant evaporation early in the experiment, prior to the complete absorption of the skin surface permeant into the SC. This requires a greater rate of permeant evaporation in Model A, and therefore a higher wind velocity.

### 4.4. Relative Predictive Accuracy of Models A and B

Figure 1 shows a wide variety in the experimentally measured dermal absorption profiles among IVPTs used to calibrate Model B: benzophenone showed a mass balance that varied depending on the solvent (73% in PBS, 46% in ethanol), propylparaben showed highly varying, solvent-dependent, levels of dermal delivery (76% in PBS, 11% in ethanol) and hydrocortisone showed little difference in dermal absorption between the two solvents, with low SC accumulation and dermal delivery in both vehicles. As seen in Figure 1, this variety in dermal absorption was well-captured by Model B after optimization.

For IVPTs conducted in a PBS solvent, Model A yielded a lower cumulative dermal delivery error than Model B (Table 5). Nevertheless, in this set of predictions, the performance of the two models was similar, with Model A and Model B predicting dermal delivery in 22/26 and 18/26 experiments within one order of magnitude of the measured mean value. On the other hand, Model B yielded significantly more accurate predictions among IVPTs conducted in ethanol. The total weighted sum of squares of errors ($E_{Total}^{2}$ in Table 5), show that Model B yielded a 1.5-fold improvement in overall error compared to Model A among compounds tested in PBS solvents and a corresponding 19-fold improvement among compounds tested in the ethanol solvent.

### 4.5. Application of the Learned Model B Parameters to Future Dermal Absorption Estimates

The calibrated depth of the deposition layer in the SC, $\eta_{dep}$ (given in Table 3), may be used in future predictions of dermal absorption using Model B. However, both the wind velocity u, and the vehicle evaporation rate $k_{evap-veh}$, are liable to vary significantly with ambient conditions. The calibrated values of these quantities in Table 3 reflect the Franz cell ambient conditions of the experiments reported in [5] that were not conducted in a fume hood. Nevertheless, the calibrated $k_{evap-veh}$ values give an indication of the timescale in which aqueous and ethanol solvents may be expected to completely evaporate in non-occluded conditions. In [5], the vehicle volumes were 10 µL, and these vehicles were applied to skin samples of area 1 cm^2^, yielding a vehicle thickness of 0.01 cm. The vehicle evaporation rates in Table 3 therefore translate to a drying time of 43 s for the ethanol solvent and 109 s for the aqueous solvent.

### 4.6. Implications for Risk Assessment

In the absence of in vivo human dermal absorption data, a risk assessment that includes IVPT assessments of dermal absorption can provide estimates of the potential absorption of a topically applied chemical [23]. In ideal situations, experimental dermal absorption data would be generated under conditions closely mimicking the ‘in-use’ exposure condition that is being evaluated for therapeutic efficacy or toxicological risk. However, dermal absorption studies are resource-intensive, and the extrapolation of the results may be limited by differences between the experimental conditions of the study protocol and the intended in vivo scenario. Here, in silico/computational models of skin absorption can aid decision making in chemical risk assessment. In cases where IVPT data is available for the compound of interest, a typical in vitro to in vivo workflow for estimating internal (systemic) exposure would involve optimizing a dermal absorption model to IVPT data and then simulating the calibrated model to estimate in vivo skin permeation. For such purposes, Models A and B constitute priors that can be optimized to data from small-dose IVPTs conducted using volatile vehicles.

The choice of which of the two models to use ultimately depends on the requirements of the risk assessment. Often, the aim is simply to obtain an upper bound on dermal absorption, for which case Model A is more likely to provide a worst-case estimate of exposure due to its tendency to over-predict dermal delivery. In other studies, however, it may be necessary to build an accurate understanding of permeant disposition within the different skin compartments over time. For example, it may be necessary to estimate stratum corneum accumulation as repeated dermal applications can result in a permeant’s build-up within the SC, followed by slow release into the bloodstream [24]. In such a scenario, Model B is more likely to provide an accurate picture of long-term SC accumulation given its lower overall estimation errors (Table 5).

### 4.7. Future Research Directions: Workflow Development for Formulation Selection

In future work, the approach adopted in this study to learn vehicle-specific properties can be generalized to build predictive models of dermal absorption from complex formulations such as creams, lotions, or gels in which active pharmaceutical ingredients (APIs) are encapsulated in droplets or nanoparticles [19,25,26]. In such formulations, the loss of volatile vehicle components can induce phase transformations in the applied preparation, leading to changes in the permeant absorption rate [6,8,27] in a way that parallels the evaporation process in Model B.

**Figure 7:**
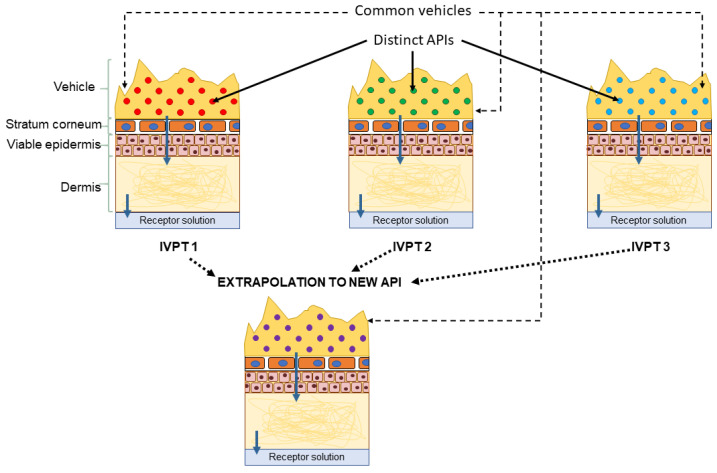
Proposed extrapolation workflow: the vehicle-specific parameters are learned from IVPTs conducted using a common vehicle but distinct active pharmaceutical ingredients (APIs). The learned parameters can inform future estimates of dermal absorption of novel APIs from the same vehicle.

As illustrated in Figure 7, measurements of dermal disposition by multiple APIs applied in a common, complex, formulation of interest, can be used to train the dermal model to predict skin penetration by a novel, previously untested, API when applied via the same formulation. Such an extrapolation would consist of three steps: first the model would be trained using the IVPT measurements to learn mappings, specific to the formulation of interest, between, on the one hand, the physical/chemical descriptors of a given API and, on the other hand, quantities that govern the transport of the API into and across the stratum corneum, such as the vehicle/SC partition coefficient and the stratum corneum diffusivity. These mappings would quantify the aggregate effect of the formulation on the transport parameters. In the next step, the learned mappings can be used to infer the transport parameters for the novel API from its physical/chemical descriptors, when applied in the formulation of interest. Finally, estimates of the dermal absorption of the novel API, when applied in the formulation of interest, can be generated by simulating the model using the inferred transport parameters.

This workflow can be applied to a wide selection of formulations, resulting in a library of formulation-specific mappings that can be used to generate estimates of dermal absorption from multiple vehicles for a novel API based on its physical and chemical descriptors. Such a library can henceforth be used to select the most appropriate formulation for a given, novel API to inform and prioritize a testing strategy during product development and safety evaluation.

### 4.8. Limitations

Among the limitations of Model B is that common evaporation rates are proposed for all IVPTs conducted using a given solvent. This assumption neglects possible interactions between the solvent and the permeant that can cause them to mutually alter their volatilities. In addition, the deposition layer proportion of the SC, $\eta_{dep}$, provides an empirical quantification of the aggregate effect of multiple fast-timescale processes that impact permeant absorption immediately following topical application. We have optimized this quantity specifically for a PBS and an ethanol solvent. For other solvents, estimating $\eta_{dep}$ would require further data from IVPTs conducted using those vehicles.

## 5. Conclusions

In this study, we have developed a mechanistic model of permeant transport kinetics into the stratum corneum that accounts for the effects of vehicle volatility and permeant crystallization on the skin surface upon evaporation of the solvent. Assuming the vehicle evaporation rate and the deposition layer depth to be vehicle-specific, the model parameters representing these quantities were calibrated using in vitro skin permeation data measuring the disposition of multiple permeants applied to skin in both saline and ethanol solvents. The optimized values indicate that the ethanol vehicle yields a greater deposition layer depth than the saline solvent, thus demonstrating the former’s penetration enhancing effect. With respect to an earlier model that does not account for vehicle-specific effects, the trained model demonstrated significant improvements in predicting the dermal disposition of thirty-one compounds previously tested in vitro. The proposed model structure can therefore provide a basis for future in vitro to in vivo extrapolations involving volatile vehicles. In addition, the optimization method adopted in this paper can be generalized into a workflow for learning vehicle-specific effects for formulations that are more complex than the solvents considered in this work. Application of this workflow to multiple formulations can yield a library of formulation-specific information from which to select vehicle candidates that yield target levels of dermal delivery for optimal efficacy and safety evaluation.

## Figures and Tables

**Table 1:** Compounds tested in PBS solvents in [5] and selected for model assessment.

1-4-Phenylenediamine	Caffeine
2-5-Diaminotoluene sulfate	Cinnamic acid
2-Amino-3-methylimidazo [4-5-f]quinoline	Cinnamyl Alcohol
2-Aminophenol	Cyclophosphamide monohydrate
2-Nitro-1-4-phenylenediamine	Diethanolamine
4-Amino-3-nitrophenol	HC Red No3
4-Nitro-1-2-Phenylenediamine	Hydroquinone
6-Methylcoumarin	Ibuprofen
7-Ethoxycoumarin	Methylparaben
Anisyl alcohol	Propylparaben (experiment 2 in [5])
Basic Red 76 (Chloride salt)	Resorcinol
Benzoic Acid	Thioglycolic acid
Benzylidene acetone	Vanillin

**Table 2:** Compounds tested in an ethanol solvent in [5] and selected for model assessment.

2-Acetylaminofluorene
4-Bromophenyl isocyanate
Testosterone
Tetramethyl thiuram disulfide
Triclosan

**Table 3:** Summary of calibration results for Models A and B.

Model	Parameter	Description	**Calibrated Value**
A	$u_{A}$	Wind velocity	43 cm/min
B	$u_{B}$	Wind velocity	0.68 cm/min
B	$\eta_{dep}^{pbs}$	Deposition layer proportion of SC under PBS vehicle	0.03
B	$\eta_{dep}^{ethanol}$	Deposition layer proportion of SC under ethanol vehicle	0.05
B	$k_{evap-veh}^{pbs}$	Evaporation rate of PBS vehicle	0.0055 cm/min
B	$k_{evap-veh}^{ethanol}$	Evaporation rate of ethanol vehicle	0.014 cm/min

**Table 4:** Summary of measurements from Hewitt et al. [5] and simulations of Models A and B of skin wash, SC accumulation and dermal delivery at 24 h for IVPTs selected for model assessment.

**Solvent**	**Compound**	**Dose (µg/cm^2^)**	Skin Washat t = 24 h (µg/cm^2^)				Stratum Corneum Accumulation at t = 24 h (µg/cm^2^)				Cumulative Dermal Delivery at t = 24 h (µg/cm^2^)			
			Observed		Predicted		Observed		Predicted		Observed		Predicted	
			**Mean** $y_{SW}$	**SD** $\sigma_{SW}$	**Model A** ${\bar{y}}_{SW}$	**Model B** ${\bar{y}}_{SW}$	**Mean** $y_{SC}$	**SD** $\sigma_{SC}$	**Model A** ${\bar{y}}_{SC}$	**Model B** ${\bar{y}}_{SC}$	**Mean** $y_{DD}$	**SD** $\sigma_{DD}$	**Model A** ${\bar{y}}_{DD}$	**Model B** ${\bar{y}}_{DD}$
PBS	6-Methylcoumarin	4.21	0.14	0.08	0.01	3.62	0.01	0	0.04	0.01	4.06	0.14	3.59	0.47
	Vanillin	3.3	0.72	0.24	0	1.80	0.06	0.02	0.44	0.20	1.92	0.33	2.85	1.28
	7-Ethoxycoumarin	1.13	0.07	0.04	0	0.59	0	0	0.04	0.02	1.08	0.06	1.06	0.48
	4-Amino-3-nitrophenol	11.76	6.08	1.4	0.69	11.35	0.41	0.27	7.97	0.23	4.94	1.60	3.09	0.17
	Caffeine	1.08	0.58	0.21	0	0	0.02	0.01	1.05	1.05	0.44	0.21	0.03	0.03
	Benzoic Acid	7.92	4.33	0.61	0	4.20	0.17	0.08	0	0	2.74	0.64	7.92	3.60
	Thioglycolic acid	72.46	47.69	5.12	0	41.19	4.32	1.23	0.93	0.25	10.28	6.69	71.53	19.21
	2-5-Diaminotoluene sulfate	0.94	0.72	0.04	0	0	0.02	0.01	0.33	0.32	0.14	0.03	0.61	0.61
	Propylparaben (PBS—experiment 2)	2.51	0.75	0.15	0	0.60	0.03	0.02	0	0	1.66	0.23	2.51	1.52
	Cyclophosphamide monohydrate	47.42	39.45	6.39	23.56	45.64	1.60	1.08	23.8	1.73	4.87	5.27	0.07	0.01
	Resorcinol	97.86	16.69	5.79	0	69.81	5.13	2.96	0.01	0	72.61	8.89	97.85	27.98
	Benzylidene acetone	3.91	0.25	0.04	0	0.01	0.06	0.01	0.01	0	2.83	0.15	3.90	1.70
	Ibuprofen	2.51	1.89	0.42	0	0	0.11	0.06	0	0	0.56	0.41	2.51	2.50
	Methylparaben	3.21	0.54	0.29	0	0.99	0.01	0	0.02	0.01	2.47	0.30	3.19	1.34
	2-Aminophenol	5.85	2.18	0.93	0	3.51	0.19	0.09	0.33	0.13	2.89	0.85	5.52	2.14
	Anisyl alcohol	11.95	0.58	0.29	0.02	10.76	0.01	0.01	1.14	0.07	10.17	0.69	10.79	0.79
	Cinnamic acid	1.62	1.16	0.14	0	0.64	0.05	0.02	0	0	0.39	0.14	1.62	0.70
	2-Nitro-1-4-phenylenediamine	5.78	3.67	1.25	0.01	5.56	0.08	0.06	3.67	0.11	1.95	1.12	2.11	0.11
	Cinnamyl Alcohol	6.72	0.28	0.19	0	0.01	0	0	0	0	5.86	0.37	6.72	3.00
	Diethanolamine	10.2	9.88	0.28	0	0	0.13	0.06	7.89	7.92	0.19	0.20	2.31	2.28
	1-4-Phenylenediamine	0.91	0.49	0.06	0	0	0.09	0.03	0.43	0.42	0.17	0.06	0.48	0.48
	Hydroquinone	15.73	9.05	1.90	0	9.33	1.54	0.87	1.08	0.44	4.01	2.59	14.65	5.95
	2-amino-3-methylimidazo[4 -5-f]quinoline	3.21	2.37	0.33	0.01	2.98	0.51	0.26	1.23	0.08	0.29	0.21	1.97	0.15
	4-Nitro-1-2-Phenylenediamine	1.18	0.54	0.11	0	1.12	0.05	0.01	0.39	0.02	0.55	0.09	0.79	0.05
	Basic Red 76 (Chloride salt)	53.27	51.10	0.78	45.69	52.15	0.48	0.21	7.58	1.12	0.12	0.11	0	0
	HC Red No3	22.04	19.91	0.85	6.59	21.30	0.81	0.49	14.06	0.63	0.62	0.41	1.39	0.11
Ethanol	4-Bromophenyl isocyanate	1.24	0.90	0.14	0	0	0.21	0.10	0	0	0.03	0.02	0.05	0.04
	2-Acetyl aminofluorene	1.3	1.12	0.07	0.77	1.29	0.03	0.02	0.12	0	0.08	0.05	0.40	0.01
	Testosterone	1.64	1.41	0.13	0.01	1.35	0.07	0.03	0.27	0.05	0.08	0.06	1.36	0.24
	Tetramethyl thiuram disulfide	1.21	0.92	0.07	0	1.12	0.04	0.02	0.67	0.05	0.05	0.03	0.44	0.04
	Triclosan	1.81	1.41	0.07	0	1.30	0.22	0.07	0.12	0.03	0.08	0.02	1.69	0.48

**Table 5:** Summary of squared model errors defined in Table A4 at t=24 h summed over IVPTs selected for model assessment for PBS and ethanol solvents (see Table A4).

Solvent	Quantity	Model A	Model B
PBS	**Skin wash error** $\left(e_{SW}^{2}\right)$	2392	5343
	**Stratum corneum accumulation error** $\left(e_{sc}^{2}\right)$	51,691	28,925
	**Cumulative dermal delivery error** $\left(e_{DD}^{2}\right)$	851	1566
	**Weighted sum of squares error** $\left(E_{Total}^{2}\right)$	54,926	35,772
Ethanol	**Skin wash error** $\left(e_{SW}^{2}\right)$	764	59
	**Stratum corneum accumulation error** $\left(e_{sc}^{2}\right)$	885	14
	**Cumulative dermal delivery error** $\left(e_{DD}^{2}\right)$	7200	405
	**Weighted sum of squares error** $\left(E_{Total}^{2}\right)$	8848	478

## Data Availability

All data used in this study is available from published material cited in the manuscript.

## Appendix A. Nomenclature

**Table A1:** Nomenclature table.

Nomenclature	
$c_{sc}\left(z,t\right)$	Concentration of the permeant in the stratum corneum, at skin depth z and time t.
$c_{ed}\left(z,t\right)$	Concentration of the permeant in the epidermis, at skin depth z and time t.
$c_{de}\left(z,t\right)$	Concentration of the permeant in the dermis, at skin depth z and time t.
u	Wind velocity
$M_{0}$	Dose of permeant (mass per area).
$M_{surf}$	Permeant mass per area of skin on skin surface.
$C_{sat}$	Saturation concentration of permeant in stratum corneum.
$K_{sc}$	Partition coefficient of permeant in stratum corneum relative to water.
$D_{sc}$	Stratum corneum diffusion constant.
$K_{ed}$	Partition coefficient of permeant in viable epidermis relative to water.
$D_{ed}$	Viable epidermis diffusion constant.
$K_{de}$	Partition coefficient of permeant in dermis relative to water.
$D_{de}$	Dermis diffusion constant.
$k_{de}$	Dermis clearance rate constant.
$k_{evap-per}$	Rate of evaporation of permeant.
$k_{evap-veh}$	Rate of evaporation of vehicle.
$h_{sc}$	Stratum corneum thickness
$h_{ed}$	Epidermis thickness
$h_{de}$	Dermis thickness
$\eta_{dep}$	Proportion of stratum corneum thickness occupied by deposition layer
$y_{SW}\left(t\right)$	Measured mean skin wash at time t
$y_{SC}\left(t\right)$	Measured mean stratum corneum accumulation at time t
$y_{DD}\left(t\right)$	Measured mean cumulative dermal delivery at time t
$y_{MB}\left(t\right)$	Measured mean mass balance at time t
$\sigma_{SW}\left(t\right)$	Measured standard deviation in skin wash at time t
$\sigma_{SC}\left(t\right)$	Measured standard deviation in stratum corneum accumulation at time t
$\sigma_{DD}\left(t\right)$	Measured standard deviation in cumulative dermal delivery at time t
$\sigma_{MB}\left(t\right)$	Measured standard deviation in mass balance at time t

## Appendix B. Mathematical Details

**Table A2:** Mathematical details of Models A and B.

Model A	Model B
Initial conditions	
$M_{surf}\left(0\right)=\max\left(0,M_{0}-\eta_{dep}h_{sc}C_{sat}\right)$	
$c_{sc}\left(z,0\right)=\min\left(\frac{M_{0}}{\eta_{dep}h_{sc}},C_{sat}\right),\;0\leq z<\eta_{dep}h_{sc}$	
$c_{sc}\left(z,0\right)=0,\;\eta_{dep}h_{sc}\leq z<h_{sc}$	
$c_{ed}\left(z,0\right)=0,\;h_{sc}\leq z<h_{sc}+h_{ed}$	
$c_{de}\left(z,0\right)=0,\;h_{sc}+h_{ed}\leq z<h_{sc}+h_{ed}+h_{de}$	
**Boundary conditions**	
${\dot{M}}_{surf}=-D_{sc}\frac{\partial c_{sc}\left(\eta_{dep}h_{sc},t\right)}{\partial z}-k_{evap-per}$ for $M_{surf}>0$ $D_{sc}\frac{\partial c_{sc}\left(0,t\right)}{\partial z}=k_{evap-per}\cdot c_{sc}\left(0,t\right)/C_{sat}$ otherwise	${\dot{h}}_{v}=\{\begin{array}{c} -k_{evap-veh},\\ 0, \end{array}\begin{array}{r} h_{v}>0\\ \mathrm{otherwise} \end{array}$ ${\dot{M}}_{surf}=\{\begin{array}{rr} -k_{evap-per}-D_{sc}{\frac{\partial c_{sc}}{\partial z}}_{|z=0}, & h_{v}>0\;\mathrm{and}\;M_{surf}>0\\ -k_{evap-per}, & h_{v}=0\;\mathrm{and}\;M_{surf}>0\\ 0, & \mathrm{otherwise} \end{array}$ $D_{sc}{\frac{\partial c_{sc}}{\partial z}}_{|z=0}=\{\begin{array}{rr} k_{evap-per}\cdot c_{sc}\left(0,t\right)/C_{sat}, & \;M_{surf}=0\\ 0, & h_{v}=0\;\mathrm{and}\;M_{surf}>0 \end{array}$
$\frac{c_{sc}\left(h_{sc},t\right)}{K_{sc}}=\frac{c_{ed}\left(h_{sc},t\right)}{K_{ed}}$	
$D_{sc}\frac{\partial c_{sc}\left(h_{sc},t\right)}{\partial z}=D_{ed}\frac{\partial c_{ed}\left(h_{sc},t\right)}{\partial z}$	
$\frac{c_{ed}\left(h_{sc}+h_{ed},t\right)}{K_{ed}}=\frac{c_{de}\left(h_{sc}+h_{ed},t\right)}{K_{de}}$	
$D_{ed}\frac{\partial c_{ed}\left(h_{sc}+h_{ed},t\right)}{\partial z}=D_{de}\frac{\partial c_{de}\left(h_{sc}+h_{ed},t\right)}{\partial z}$	
$\frac{\partial c_{de}\left(h_{sc}+h_{ed}+h_{de},t\right)}{\partial z}=0$ (in vivo case)	
$c_{de}\left(h_{sc}+h_{ed}+h_{de},t\right)=0$ (in vitro case)	
**Dynamics**	
$\frac{\partial c_{sc}\left(z,t\right)}{\partial t}=\frac{\partial^{2}c_{sc}\left(z,t\right)}{\partial z^{2}},$ for$\;0\leq z<h_{sc}$	
$\frac{\partial c_{ed}\left(z,t\right)}{\partial t}=\frac{\partial^{2}c_{ed}\left(z,t\right)}{\partial z^{2}},$ for$\;h_{sc}\leq z<h_{sc}+h_{ed}$	
$\frac{\partial c_{de}\left(z,t\right)}{\partial t}=\frac{\partial^{2}c_{de}\left(z,t\right)}{\partial z^{2}}-k_{de}c_{de}\left(z,t\right),\;$with $k_{de}>0$ (in vivo case), $k_{de}=0$ (in vitro case) for $h_{sc}+h_{ed}\leq z<h_{sc}+h_{ed}+h_{de}$	

**Table A3:** Definition of model estimates of experimentally measurable quantities at time t.

Quantity	Definition
Skin wash	${\bar{y}}_{SW}\left(t\right)=M_{surf}\left(t\right)$
Stratum corneum accumulation	${\bar{y}}_{SC}\left(t\right)=\int_{z=0}^{z=h_{sc}}c_{sc}\left(z,t\right)dz$
Cumulative dermal delivery	${\bar{y}}_{DD}\left(t\right)=\int_{z=h_{sc}}^{z=h_{sc}+h_{ed}}c_{ed}\left(z,t\right)dz+\int_{z=h_{sc}+h_{ed}}^{z=h_{sc}+h_{ed}+h_{de}}c_{de}\left(z,t\right)dz+\int_{\tau =0}^{\tau =t}D_{de}{\frac{\partial c_{de}\left(z,\tau \right)}{\partial z}}_{|z=h_{sc}+h_{ed}+h_{de}}d\tau$
Mass balance as a percentage of dose	${\bar{y}}_{MB}\left(t\right)=100\times \left({\bar{y}}_{SW}+{\bar{y}}_{SC}+{\bar{y}}_{DD}\right)/M_{0}$

**Table A4:** Definition of measures of model error. See Table A3 for definitions of model estimates.

Quantity	Definition
Skin wash error at time t	$e_{SW}\left(t\right)=\left(y_{SW}\left(t\right)-{\bar{y}}_{SW}\left(t\right)\right)/\sigma_{SW}\left(t\right)$
Stratum corneum accumulation error at time t	$e_{SC}\left(t\right)=\left(y_{SC}\left(t\right)-{\bar{y}}_{SC}\left(t\right)\right)/\sigma_{SC}\left(t\right)$
Cumulative dermal delivery error at time t	$e_{DD}\left(t\right)=\left(y_{DD}\left(t\right)-{\bar{y}}_{DD}\left(t\right)\right)/\sigma_{DD}\left(t\right)$
Root sum of squares error at time t	$E_{Total}\left(t\right)={\left({\left(e_{SW}\left(t\right)\right)}^{2}+{\left(e_{SC}\left(t\right)\right)}^{2}+{\left(e_{DD}\left(t\right)\right)}^{2}\right)}^{\frac{1}{2}}$
Mass balance error at time t	$E_{MB}\left(t\right)=\left(y_{MB}\left(t\right)-{\bar{y}}_{MB}\left(t\right)\right)/\sigma_{MB}\left(t\right)$

Log-likelihood functions used for model optimization.

Optimization of Model A maximized the likelihood function (with constant terms omitted):

$$\log L_{A}\left(u_{A}|y_{MB},t\right)=-\underset{i}{\sum }E_{MB}^{2}{}_{i}\left(u_{A},t\right)\;$$

where:

- $E_{MB}{}_{i}\left(u_{A},t\right)=E_{MB}$ as defined in Table A4 for the ith IVPT evaluated at time t=24 *h.*
- for the ith IVPT, the error $E_{MB}$ was evaluated using:
  ○ the model estimates for IVPT $i,\;{\bar{y}}_{MB}\left(t\right)$ at time t=24 *h*, generated by Model A using $u=u_{A}$, and,
  ○ $y_{MB}\left(t\right)$ corresponding to the mass balance of IVPT i at time t=24 h as measured in Hewitt et al. [5].

Optimization of Model B maximized the likelihood (with constant terms omitted):

$$\begin{array}{c} \log L_{B}(u_{B},\eta_{dep}^{pbs}\;,\eta_{dep}^{ethanol},k_{evap-veh}^{pbs},\;k_{evap-veh}^{ethanol}|y_{SW},y_{SC},\;y_{DD},t)\\ =-\underset{i}{\sum }E_{Total}^{2}{}_{i}(u_{B},\eta_{dep}^{pbs}\;,\eta_{dep}^{ethanol},k_{evap-veh}^{pbs},k_{evap-veh}^{ethanol},t) \end{array}$$

where:

- $E_{Total}{}_{i}\left(u_{B},\eta_{dep}^{pbs}\;,\eta_{dep}^{ethanol},k_{evap-veh}^{pbs},k_{evap-veh}^{ethanol},t\right)=E_{Total}$ as defined in Table A4 for the ith IVPT evaluated at time t=24 h,
- for IVPT i*,* the error $E_{Total}$ was evaluated using:
  ○ the model estimates for IVPT i*,* ${\bar{y}}_{SW}\left(t\right),{\bar{y}}_{SC}\left(t\right),{\bar{y}}_{DD}\left(t\right)$ at time t=24 h, generated by Model B using
    ▪ $u=u_{B}$*,*
    ▪ $\eta_{dep}=\eta_{dep}^{pbs}\;$and $k_{evap-veh}=k_{evap-veh}^{pbs}$ for PBS solvent IVPTs, and,
    ▪ $\eta_{dep}=\eta_{dep}^{ethanol}$ and $k_{evap-veh}=k_{evap-veh}^{ethanol}$ for ethanol solvent IVPTs

## Appendix C. Assessment of Model A Deposition Layer Capacity

**Table A5:** Dose and Model A deposition layer capacities for all compounds tested in Hewitt et al. [5].

Compound	Dose (µg/cm^2^)	$\mathrm{SC}/\mathrm{Water}\;\mathrm{Partition}\;\mathrm{Coefficient}\;K_{sc/w}$	$\mathrm{SC}\;\mathrm{Saturation}\;\mathrm{Concentration}C_{sat}\;(\text{µ}g/{\mathrm{cm}}^{3})$	$\mathrm{Deposition}\;\mathrm{Layer}\;\mathrm{Capacity}M_{sat}\;(\text{µ}g/{\mathrm{cm}}^{2})$	**Skin Wash (µg/cm^2^)**	**Deposition Layer Capacity > Dose?**
1-4-Phenylenediamine	0.91	3.75	31,035.56	4.03	0.49	Yes
2-4-Dichloroacetophenone	0.78	19.75	12,439.82	1.62	0.0048	Yes
2-4-Dinitrochlorobenzene	4.26	18.28	7492.78	0.97	0.44	No
2-5-Diaminotoluene sulfate	0.94	5.01	39,584.87	5.15	0.72	Yes
2-Acetyl aminofluorene (ethanol solvent)	1.3	42.00	168.00	0.02	1.12	No
2-amino-3-methylimidazo[4-5-f]quinoline	3.21	11.10	5880.38	0.76	2.37	No
2-aminophenol	5.85	6.45	129,005.20	16.77	2.18	Yes
2-Nitro-1-4-phenylenediamine	5.78	6.11	5861.87	0.76	3.67	No
4-Amino-3-nitrophenol	11.76	5.68	11,301.56	1.47	6.08	No
4-Aminophenol	4.48	4.56	3466.24	0.45	3.71	No
4-Bromophenyl isocyanate (ethanol solvent)	1.24	61.92	2167.30	0.28	0.9	No
4-Chloroaniline	5.08	14.22	46,230.42	6.01	1.94	Yes
4-Chlorobutyric acid	66.36	10.04	825,457.00	107.31	54.83	Yes
4-Methylvaleric acid	20.3	15.85	134,428.20	17.48	3.08	No
4-Nitro-1-2-Phenylenediamine	1.18	7.58	1590.91	0.21	0.54	No
4-Tolunitrile	1.89	17.20	13,588.19	1.77	0.043	No
6-Methylcoumarin	4.21	15.06	7381.77	0.96	0.135	No
7-Ethoxycoumarin	1.13	20.22	15,769.40	2.05	0.066	Yes
Acetophenone	10.27	11.95	105,894.70	13.77	0.2	Yes
alpha-Methyl-1-3-benzodioxole- 5-propionaldehyde	2.63	23.98	8151.58	1.06	0.36	No
Anisyl alcohol	11.95	8.71	17,415.46	2.26	0.58	No
Basic Red 76 (Chloride salt)	53.27	3.71	33,537.68	4.36	51.1	No
Benzoic Acid	7.92	14.64	65,135.30	8.47	4.33	Yes
Benzophenone (ethanol solvent)	0.99	44.66	9825.68	1.28	0.21	Yes
Benzophenone (PBS solvent)	1.28	44.66	9825.68	1.28	0.056	No
Benzyl bromide (acetone solvent)	9.12	34.52	252,705.50	32.85	0.23	Yes
Benzylidene acetone	3.91	16.94	36,091.70	4.69	0.25	Yes
Caffeine	1.08	4.28	74,864.29	9.73	0.58	Yes
Cinnamic acid	1.62	17.73	14,004.37	1.82	1.16	Yes
Cinnamyl Alcohol	6.72	15.51	61,724.10	8.02	0.28	Yes
Cyclophosphamide monohydrate	47.42	6.49	52,502.84	6.83	39.45	No
Diethanolamine	10.2	2.03	2,029,273.00	263.81	9.88	Yes
Diethyleneglycol butyl ether	64.11	6.22	3,080,250.00	400.43	0.66	Yes
Diethylmaleate	24.08	18.70	288,926.00	37.56	3.12	Yes
Dimethyl fumarate	6.24	5.07	405,633.60	52.73	0.31	Yes
Dimethyl phthalate	1.31	12.12	38,530.15	5.01	0.21	Yes
ethylhexyl acrylate	6.63	133.87	170,015.20	22.10	1.67	Yes
Eugenol	6.23	19.75	63,383.82	8.24	0.29	Yes
Geraniol (ethanol solvent)	2.92	67.95	23,782.20	3.09	0.77	Yes
Geraniol (PBS solvent)	2.36	67.95	23,782.20	3.09	0.13	Yes
HC Red No3	22.04	6.03	22,018.61	2.86	19.91	No
Hydrocortisone (ethanol solvent)	5.41	12.20	11,833.66	1.54	4.42	No
Hydrocortisone (PBS solvent)	5.53	12.20	11,833.66	1.54	4.95	No
Hydroquinone	15.73	6.33	375,682.60	48.84	9.05	Yes
Ibuprofen	2.51	113.73	46,630.21	6.06	1.89	Yes
Isoeugenol	3.52	38.77	23,262.68	3.02	0.6	No
Methyl Methane sulfonate	24.83	3.35	1,775,874.00	230.86	2.14	Yes
Methylisothiazolinone	13.62	2.79	1,497,644.00	194.69	0.83	Yes
Methylparaben	3.21	15.62	36,555.72	4.75	0.54	Yes
Naphthalene (ethanol solvent)	0.93	50.70	1520.88	0.20	0.114	No
Nitrobenzene	3.7	14.43	67,672.38	8.80	0.28	Yes
Propylparaben (ethanol solvent)	2.96	38.77	20,160.99	2.62	2.16	No
Propylparaben (PBS solvent-experiment 2)	2.51	38.77	20,160.99	2.62	0.75	Yes
Propylparaben (PBS solvent-experiment 1)	2.42	38.77	20,160.99	2.62	0.43	Yes
Resorcinol	97.86	7.21	3,630,053.00	471.91	16.69	Yes
Testosterone (ethanol solvent)	1.64	51.80	6734.57	0.88	1.41	No
Tetramethyl thiuram disulfide (ethanol solvent)	1.21	13.26	1590.72	0.21	0.917	No
Thioglycolic acid	72.46	4.70	2,214,796.00	287.92	47.69	Yes
trans-Cinnamaldehyde	2.42	14.96	20,939.01	2.72	0.1	Yes
Triclosan (ethanol solvent)	1.81	364.45	14,577.90	1.90	1.41	Yes
Vanillin	3.3	9.35	79,078.40	10.28	0.72	Yes
